# A novel phenolic propanediamine moiety-based lung-targeting therapy for asthma

**DOI:** 10.1080/10717544.2018.1472675

**Published:** 2018-05-21

**Authors:** Jianbo Li, Yang Yang, Didi Wan, Youmei Peng, Jinjie Zhang

**Affiliations:** a Institute of Medical and Pharmaceutical Sciences, Zhengzhou University, Zhengzhou, PR China;; b School of Pharmaceutical Sciences, Zhengzhou University, Zhengzhou, PR China;; c Key Laboratory of Targeting Therapy and Diagnosis for Critical Diseases, Zhengzhou, PR China;; d Collaborative Innovation Center of New Drug Research and Safety Evaluation, Zhengzhou, PR China

**Keywords:** Asthma therapy, phenolic propanediamine, lung-targeting, Rhein, small ligand

## Abstract

Asthma is one of the most prevalent chronic inflammatory diseases of lung. Current asthma therapy using inhaled corticosteroid often results in undesired treatment outcome due to poor compliance and drugs’ lack of tissue specificity. N,N,N’-trimethyl-N’-(2-hydroxyl-3-methyl-5-^123^Iiodobenzyl)-1,3-propanediamine (HIPD), a phenolic propanediamine derivative, has been used as an imaging agent for localized pulmonary diseases. Inspired by this, N,N,N’-trimethyl-N’-(4-hydroxyl-benzyl)-1,3-propanediamine (TPD), a new HIPD analog, was proposed as a lung-targeting ligand and covalently conjugated to an anti-inflammatory compound Rhein for asthma therapy. Cellular uptake efficiency of TPD-Rhein by A549 cells was significantly enhanced compared with Rhein. The enhanced cellular uptake was mainly mediated by organic cation transporters (OCTs) in an active manner, showing concentration- and energy-dependent. After systemic administration in rats, TPD-Rhein specifically distributed to lungs, displaying the highest *C*_max_ and AUC_0−_*_t_* values of all tested tissues and resulting in a 13-fold increase in *C*_max_ and a 103-fold increase in AUC_0−_*_t_* for lung compared with Rhein. Also, TPD-Rhein remarkably decreased serum histamine levels, serum IL-5 levels as well as bronchoalveolar lavage fluid IL-5 levels in lungs of asthmatic rats challenged by ovalbumin (OVA). Accordingly, histological examinations demonstrated that TPD-Rhein attenuated lung inflammation in rats, with no apparent toxicity against major organs. Together, phenolic propanediamine-based lung-targeting approach represents an efficient and safe strategy for asthma therapy.

## 1. Introduction

Asthma is a chronic inflammatory disease of lung marked by reversible airway edema, airway hyperresponsiveness and increased mucus secretion (Barnes, 2010). As the most common chronic disease, asthma currently affects over 300 million people globally, imposing a substantial burden on the health-related quality of life of patients (Long, 2011). To date, inhaled corticosteroid remains the first-line treatment for asthma (Spangler, 2012). Nevertheless, because of poor compliance and low delivery efficiency, the regular inhaled therapy is difficult to exert its desired efficacy (Inhaler Error Steering et al., 2013). In addition, the continuous systemic exposure of corticosteroids after inhalation can be associated with notorious systemic side effects, due to the drugs’ lack of tissue specificity. These limitations related to inhaled corticosteroids might necessitate alternative asthma therapies with improved efficacy and minimized side effects.

Recent advances in lung-targeted strategies *via* systemic administration have shown great promise to satisfy the unmet needs in asthma therapy (Cao et al., 2011; Li et al., 2017). Nanoscale carriers show promising results, while the issues such as complex structures and poor stability remain unresolved (Vega-Villa et al., 2008). Macromolecular carriers-based lung-targeting strategies such as antibody- and peptide drug-conjugated systems have also been successfully developed, but their application is often limited by insufficient potency and immunogenicity (Balyasnikova et al., 2002; Teicher & Chari, 2011). Thus, small ligand-based prodrug approach with well-determined structures, low immunogenicity, and high targeting efficiency may help overcome existing challenges.

N,N,N’-trimethyl-N’-(2-hydroxyl-3-methyl-5-^123^Iiodobenzyl)-1,3-propanediamine (^123^I-labeled HIPD), a phenolic propanediamine derivative, has been used as an imaging agent to detect localized pulmonary diseases (Shih et al., 1988; Miniati et al., 1996). It is important to note that the propanediamine moiety of HIPD is a linear moiety with two tertiary amines. Meanwhile, it has been proven that lung is a site for accumulation and sequestration of several lipophilic amines (Gazdar et al., 1980). Thus, the phenolic propanediamines-based moiety may act as a potential lung-targeted ligand and benefit lung-targeted drug delivery. Inspired by this, we hypothesized that a new HIPD analog (N,N,N’-trimethyl-N’-(4-hydroxyl-benzyl)-1,3-propanediamine, TPD) might deliver anti-inflammatory agents specifically to the lung with high efficiency and low toxicity. Rhein (4,5-dihydroxyanthraquinone-2-carboxylic acid), one of the major bioactive components of Rheum palmatum, is well known for its anti-inflammatory and anti-allergic activity (Gao et al., 2014; Singh et al., 2012). However, the therapeutic efficacy of Rhein for asthma *in vivo* was compromised by its poor lung selectivity and therefore Rhein was selected as the model drug (Singh et al., 2012). This study was designed to directly validate the hypothesis.

The conjugate (TPD-Rhein) was thus synthesized and investigated *in vitro* and *in vivo*. As expected, TPD-Rhein showed significantly enhanced cellular uptake by human alveolar epithelial cells (A549) which was mainly attributed to organic cationic transporters. Tissue distribution, lung bioavailability, and therapeutic efficacy of TPD-Rhein were evaluated and compared with those of Rhein which demonstrated that TPD would be an excellent candidate ligand to achieve lung-specific dug delivery. This is the first use of a phenolic propanediamine-based moiety as a lung-specific targeting drug ligand for asthma therapy.

## 2. Materials and methods

### 2.1. Materials

Rhein was purchased from Rongsheng Biological Co. Ltd. (Xian, China). 4-(Benzyloxy) benzylchloride, N,N,N’-trimethylpropane-1, 3-diamine, taurocholic acid sodium salt hydrate (TAS), and 3-(4,5-dimethylthiazol-2-yl)-2,5-diphenyltetrazolium bromide (MTT) were commercially purchased from Sigma-Aldrich (St. Louis, MO). All other chemicals were of analytical or high-performance liquid chromatography grade. Thin-layer chromatography (silica gel GF254) was used to detect spots by UV radiation. Purification of the desired compounds was achieved by column chromatography on silica gel. Chemical shifts were expressed in parts per million (ppm, δ units). Coupling constants were in units of Hertz (Hz). MS spectroscopy was evaluated on Bruker microTOF-QII. ^1^H-NMR (nuclear magnetic resonance) analyses were performed by AMX-400 Bruker Spectrometer (Bruker BioSpin, Karlsruhe, Germany).

### 2.2. Cell culture and animals

Male Wistar rats (body weight: 200 ± 20 g) were supplied by the Experimental Animal Center of Zhengzhou University (Zhengzhou, China). Rats were maintained in a germ-free environment and allowed free access to food and water for at least one week before experiments. All animal experiments were approved by the Institutional Animal Care and Ethics Committee of Zhengzhou University, according to the requirements of the National Act on the use of experimental animals (PR China). A549 cells (human pulmonary alveolar epithelial cells), L929 cells (mouse fibroblast cells), and Hela cells (human carcinoma cervical cells) were obtained from ATCC (Manassas, VA). The cells were cultured in DMEM with high glucose (GIBCO, Big Cabin, OK) supplemented with 20% fetal bovine serum and 1% penicillin/streptomycin. Routinely, cells were maintained at 37 °C in a humidified atmosphere containing 5% CO_2_, and the cell medium was changed every other day.

### 2.3. Synthesis and characterization of Rhein conjugate (TPD-Rhein)

4-(Benzyloxy) benzylchloride (0.67 g, 2.9 mmol) and N,N,N′-trimethyl-1, 3-propanediamine (0.5 g, 4.3 mmol) were dissolved in 100 mL acetonitrile. The reaction mixture was refluxed at 85 °C for 3 h. Afterwards, the solvent was removed under reduced pressure to provide an orange-colored oil. The oily residue was added 50 mL dichloromethane (CH_2_Cl_2_) and washed several times with brine to remove hydrophilic hybrids. The organic phase was dried over anhydrous sodium sulfate and evaporated under reduced pressure to give the crude product. The crude product was hydrogenated in ethanol (20 mL) in the presence of 10% palladium on charcoal (50 mg) in an initial hydrogen pressure of 34 MPa for 24 h. The product was purified by column chromatography on silica gel with CH_2_Cl_2_/MeOH (MeOH/CH_2_Cl_2_=1:10) to yield the desired compound TPD.

To a solution of TPD (0.18 g, 0.83 mmoL) and Rhein (0.20 g, 0.70 mmol) in CH_2_Cl_2_ 50 mL, HATU (0.32 g, 0.83 mmol) and triethylamine (0.14 mL, 1.00 mmol) were added and stirred at room temperature for 12 h. Thereafter, the solvent was washed several times with water, dried over anhydrous sodium sulfate and evaporated under reduced pressure. The final residue was purified by column chromatography on silica gel with CH_2_Cl_2_/MeOH, 15:1 (v/v) to obtain the desired compound TPD-Rhein.

### 2.4. Sample preparation and LC-MS/MS analysis

Cell lysates, tissue homogenates, and plasma samples were mixed with suitable volume of methanol to precipitate protein, respectively. The mixtures were vortexed for 5 min and centrifuged at 13,000 rpm for 10 min. Subsequently, supernatants were analyzed by liquid chromatography-tandem mass spectrometry (LC-MS/MS).

A sensitive, rapid, and accurate LC-MS/MS method was developed for the determination of Rhein and TPD-Rhein in the following studies. LC-MS/MS analysis was performed using an Agilent 1200 series RRLC system equipped with an SL autosampler, degasser and SL binary pump as well as an Agilent triple-quadruple MS. Separations were carried out using a Diamonsil ODS column (50 mm × 4.6 mm, 1.8 μm) with the corresponding guard column (ODS, 5 μm). For the determination of Rhein, the mobile phase was composed of acetonitrile and 0.1% formic acid (84:16, v/v). For TPD-Rhein, the mobile phase was adjusted to acetonitrile and 0.7% formic acid (80:20, v/v). Flow rate was 0.4 mL/min and the injection volume was 1 μL. Detection was carried out on an Agilent triple-quadruple mass spectrometer. The quantification was performed using multiple reaction monitoring (MRM) and the nebulizer gas was nitrogen. Negative and positive electrospray source ion modes were used to monitor Rhein and TPD-Rhein, respectively. MRM of m/z 284 → 267 and 488 → 267 were adopted to quantify Rhein and TPD-Rhein, respectively. The voltage of fragmentor potential was 159 eV and collision energy was 32 eV. The gas follow was 10 mL/min with temperature of 350 °C, nebulizer pressure was set at 30 psi and capillary voltage was 4 kV.

### 2.5. *In vitro* stabilities of TPD-Rhein

The solubilities of Rhein and TPD-Rhein were determined in PBS with varying pH values (pH = 2 and 7.4) by equilibrating an excess of solid compound in 5 mL of buffer at 25 °C for 12 h. The samples were filtered through a 0.22 µm Millipore filter, diluted in methanol, and analyzed by LC-MS/MS. The chemical and enzymatic stabilities of TPD-Rhein were assessed in PBS with varying pH values (pH = 2, 4.5, 6.8, and 7.4), freshly prepared rat plasma and lung homogenates (diluted with 0.9% physiological saline) from rats at 37 °C, respectively. At predetermined time intervals, samples were immediately diluted by an equal amount of methanol to precipitate protein, vortexed for 5 min and centrifuged at 13,000 rpm for 10 min. The supernatant was then subjected to LC-MS/MS. Stability studies were performed in triplicate.

### 2.6. *In vitro* cytotoxicity study

To evaluate the *in vitro* cytotoxicity of Rhein and TPD-Rhein, MTT (3-(4, 5-dimethylthiazol-2-yl)-2, 5-diphenyltetrazolium bromide) assays were performed using A549 cell lines. Briefly, A549 cells were seeded in 96-well culture plates at a density of 1 × 10^4^ cells/well and incubated at 37 °C for two days. On the day of administration, the cells were treated with different concentrations of Rhein solution, TPD-Rhein solution or TPD solution (dispersed in serum-free culture medium) for 4 h. An aliquot of 20 μL MTT solution (5 mg/mL) were added into each well and incubated at 37 °C for another 4 h. After that, all medium was removed and cells were rinsed with phosphate-buffered saline (PBS), and the formed dark blue formazan crystals in each well were dissolved in 150 μL DMSO. The absorbance of each individual well was read on a microplate reader (Thermo Scientific, Waltham, MA, Varioskan Flash) at 570 nm wavelength. Cells not exposed to samples were used as control (=100% viability).

### 2.7. Cell uptake study

The A549 cells were seeded in 6-well culture plates (5 × 10^5^ cells/well). On the second day, cells were incubated with Rhein or TPD-Rhein solution at increasing concentrations (4.7, 9.3, 18.9 and 37.4 μM, respectively, equivalent to Rhein) at 37 °C for 1 h. Cells were rinsed three times with ice-cold PBS and collected for the determination of intracellular concentration. To explore the cell delivery mechanism of TPD, the cells were precubated with indicated inhibitors for 1 h and then incubated with TPD-Rhein (18.9 μM) for another 1 h. For energy depletion, cells were exposed to Rhein or TPD-Rhein (18.9 μM) for 0.5 h at 37 °C, 4 °C, or in the presence of NaN_3_ (1 mg/mL) for 1 h. Lysine and L-arginine were substrates of basic amino acid transporters (Zhou et al., 2016). Pyrilamine was used to indicate pyrilamine-sensitive transporter (Okura et al., 2008). Spermine and Spermidine were inhibitors of the alkaline polyamine transporter (Grancara et al., 2014). Tetraethylammonium specifically blocked the organic cationic transporters (Cutler & Choo, 2011). Choline was a substrate of choline transport system (Taguchi et al., 2014). TPD was performed as a competitive cationic inhibitor. The inhibitor concentrations were 10-fold (189 μM) of the concentration of TPD-Rhein. In the end, cells were rinsed three times with ice-cold PBS and the intracellular concentrations of Rhein or TPD-Rhein were measured by LC-MS/MS. Cellular uptake was expressed as the amount (nmol/L) of Rhein or TPD-Rhein per 1 mg of total cellular protein. The total protein concentration of cell lysates was measured using the BCA protein assay kit (Pierce, Appleton, WI).

### 2.8. Pharmacokinetics and biodistribution studies

Rhein or TPD-Rhein was injected through the tail vein of rats. For each preparation and sampling time point, five rats were treated with a single dose of Rhein at the dose of 10 mg/kg or TPD-Rhein at 17.2 mg/kg (dose equivalent to 10 mg/kg Rhein), respectively. The rats were scarified at predetermined time intervals (0.083, 0.25, 0.5, 1, 2, 4, 8, 16, and 24 h) after administration. The whole blood was quickly collected into heparinized tubes and centrifuged at 5000 rpm for 5 min to obtain plasma. Then, the tissues (heart, liver, spleen, lung, kidney, and brain) were rinsed with saline, weighed and homogenized with cold physiological saline (twice the weight of tissues). For Rhein treated rats, the content of Rhein in the homogenates was measured by LC-MS/MS. For TPD-Rhein treated rats, the samples were injected to LC-MS/MS to determine the concentration of free Rhein released and TPD-Rhein, respectively. The content of TPD-Rhein in tested tissues at different time point was the sum of TPD-Rhein and free Rhein released. The biodistribution of the two drugs in each organ was normalized by the weight of the selected tissue.

The pharmacokinetic data were calculated using the DAS software version 2.0 (Mathematical Pharmacology Professional Committee of China, Shanghai, China). The relative uptake efficiency (Re) and concentration efficiency (Ce) were used to evaluate the lung-targeting properties of TPD-Rhein. The values of Re and Ce were calculated as follows:

$${\text{Re}}_{\text{lung}}\text{=}{\text{(}{\text{AUC}}_{\text{0-}t\text{,}\;\text{lung}}\text{)}}_{\text{TPD-Rhein}}/{\text{(}{\text{AUC}}_{\text{0-}t\text{,}\;\text{lung}}\text{)}}_{\text{Rhein}}$$

$${\text{Ce}}_{\text{lung}}\text{=}{\text{(}C_{\text{max,}\;\text{lung}}\text{)}}_{\text{TPD-Rhein}}/{\text{(}C_{\text{max,}\;\text{lung}}\text{)}}_{\text{Rhein}}$$

Where AUC_0−_*_t_* is the area under the drug concentration–time curve from time zero to the last sampling time and *C*_max_ is the maximum drug concentration.

### 2.9. Therapeutic effect on asthma model in rats

Wistar rats were used to evaluate the efficacy of Rhein and TPD-Rhein on asthma. Forty healthy male rats (200 ± 20 g) were randomly divided into four groups (*n* = 10). Asthma model in rats was established according to a previously reported method with slight modifications (Lu et al., 2015). Briefly, on day 0, rats were systemically sensitized with an intraperitoneal injection with 1 mL of 10% ovalbumin (OVA). Two weeks after the sensitization, the rats were exposed to aerosolized OVA for 30 min of cycling three times per week for 14 weeks. The concentration of OVA was increased from 2% for 8 weeks to 4% for remaining 6 weeks to maintain the asthma condition. From weeks 3–16, the OVA-sensitized rats in the treatment groups were given normal saline, Rhein, or TPD-Rhein intravenously before OVA inhalation, at doses of 4.5 mg/kg/day for Rhein and 7.7 mg/kg/day for TPD-Rhein (dose equivalent to free drug treatment), respectively. The rats in the normal group received with the same volume of normal saline instead. One day after the last ultrasonic atomization, all rats were anesthetized by 3% sodium pentobarbital solution at a dose of 45 mg/kg. Then, 0.5 mL of intracardiac blood of each rat was collected. The histamine level and Interleukin-5 (IL-5) level in the supernatant serum were determined using Rat HIS ELISA kit (R&D Company, Minneapolis, MN) and Rat IL-5 ELISA Kit (R&D Company, Minneapolis, MN) according to the manufacturer’s instructions, respectively. Lungs were removed from sacrificed rats and lavaged to determine the IL-5 level using the corresponding kit. In the end, lungs were fixed in 4% buffered formalin, paraffin embedded and sectioned for hematoxylin and eosin staining (H&E) for conventional histological analysis.

### 2.10. Toxicity evaluation

Twenty four hour post the last treatment of TPD-Rhein, major organs of normal group and TPD-Rhein treated group were collected and processed for routine H&E histopathologic evaluation. Injuries were observed microscopically for evidence of cellular damage and inflammation.

### 2.11. Statistical analysis

Data were analyzed using two-tailed Student’s *t*-test or one-way ANOVA followed by Turkey’s multiple comparison tests. A *p* value of less than 0.05 was considered significant.

## 3. Results

### 3.1. Synthesis, characterization, and *in vitro* stability of TPD-Rhein

**Figure 1.**
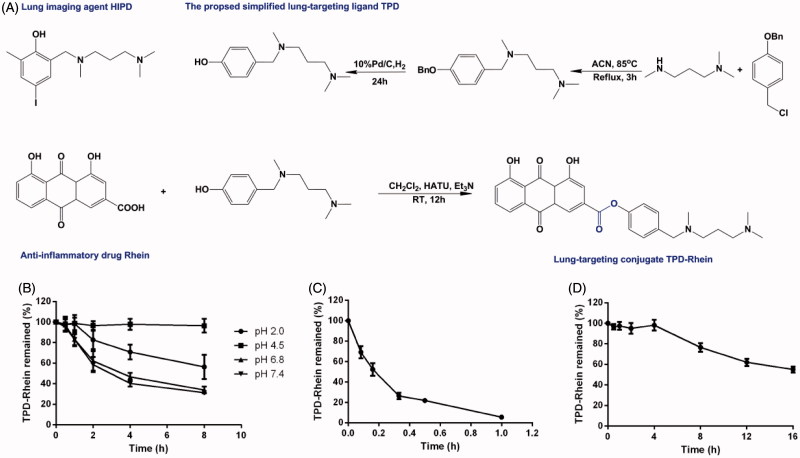
The lung-targeting ligand TPD and corresponding drug conjugate TPD-Rhein were engineered for treating asthma. (A) Based on the proposed lung-targeting moiety HIPD, TPD designed here was more feasible for the conjugation with an anti-inflammatory drug Rhein, *via* an ester bond. Degradation profiles of TPD-Rhein when incubated with PBS of different pH values (B), plasma (C) and lung homogenates (D) at 37 °C. The percentage of TPD-Rhein remained was determined by LC-MS/MS analysis and plotted against time. Data represent mean ± SD (*n* = 3).

TPD-Rhein was synthesized as outlined in Figure 1(A). Rhein was coupled to the phenol hydroxyl group on TPD through an ester bond. TPD-Rhein was obtained as an orange solid substance (23.1%). ^1^H-NMR (Figure S1) and ESI-MS confirmed the assigned structure. The detailed results were shown below.

TPD-Rhein: ^1^H-NMR (400 MHz, CDCl_3_): δ 8.59 (d, *J* = 1.6 Hz, 1H), 8.10 (d, *J* = 1.6 Hz, 1H), 7.91 (dd, *J* = 7.5, 1.2 Hz, 1H), 7.76 (t, *J* = 7.9 Hz, 1H), 7.43–7.33 (m, 3H), 7.20 (d, *J* = 8.3 Hz, 2H), 3.52 (s, 2H), 2.44 (t, *J* = 7.2 Hz, 4H), 2.33 (s, 6H), 2.23 (s, 3H), 1.75 (q, *J* = 7.4 Hz, 2H). ESI-MS: m/z [M + H]^+^: 489.19.

The aqueous solubilities of Rhein and TPD-Rhein are summarized in Table S2. The aqueous solubility of TPD-Rhein is high in acidic aqueous solutions (>10 mg/mL at pH 2.0) and low in neutral aqueous solution (<5 μg/mL). The chemical and enzymatic stabilities of TPD-Rhein were assessed in either PBS or plasma or lung homogenates. At pH 4.5, over 99% of TPD-Rhein remained stable in 0.1 M PBS (Figure 1(B)). At neutral pH (pH 6.8 and 7.4), over 40% of TPD-Rhein remained unchanged for 4 h. For enzyme stability, the remaining percentages of TPD-Rhein in plasma after 10 min (Figure 1(C)) and rat lung homogenates after 8 h (Figure 1(D)) were 53 and 76.6%, respectively.

### 3.2. *In vitro* cytotoxicity study

**Figure 2.**
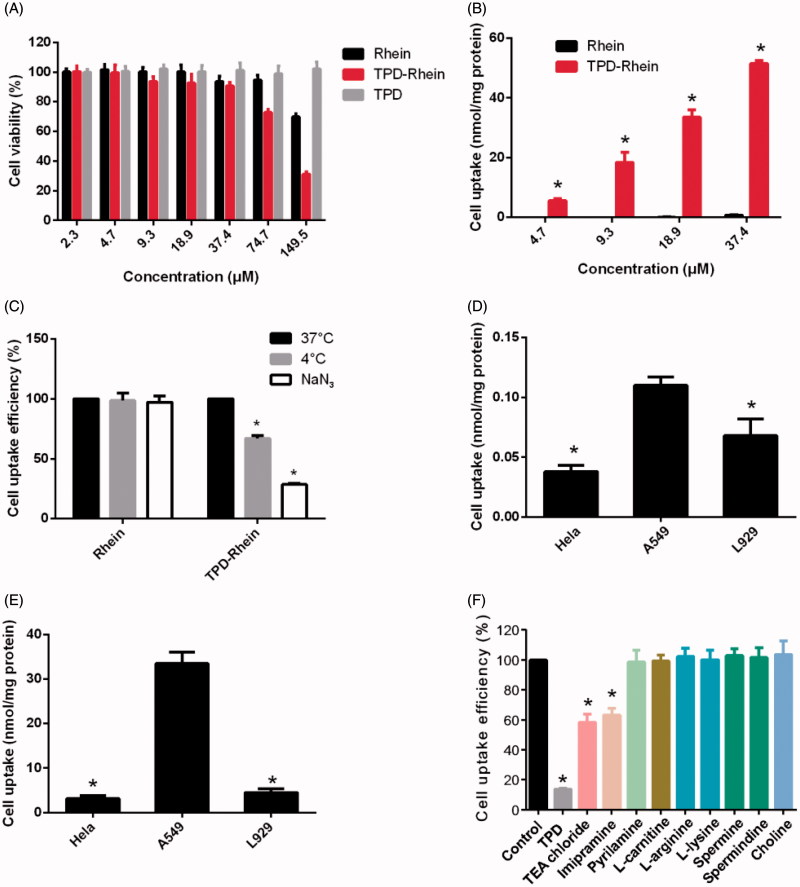
*In vitro* cellular studies of TPD-Rhein. (A) *In vitro* cytotoxicity of TPD-Rhein in A549 cells as determined by standard MTT assay. (B) Enhanced cellular uptake efficiency of TPD-Rhein in A549 cells after 1 h incubation. *p < .001 compared to Rhein group. (C) Effects of energy-depletion treatment on the cellular uptake of TPD-Rhein by A549. (D) Cell uptake efficiency of Rhein in the indicated cell lines. *p < .001, compared with cellular uptake amount of Rhein in A549 cells. (E) Cell uptake efficiency of TPD-Rhein in the indicated cell lines. *p < .001, compared with cellular uptake amount of TPD-Rhein in A549 cells. (F) Cellular uptake efficiency of TPD-Rhein in the presence of various inhibitors. TPD was used as a competitive organic cation inhibitor. The cell uptake amount without any inhibition treatment was used as control. *p < .001, compared with control group. Data represented as mean ± SD (*n* = 3).

The *in vitro* cytotoxicity of TPD-Rhein on A549 cells was shown in Figure 2(A). After 4 h treatment, TPD-Rhein showed slightly higher cytotoxicity compared with Rhein group at the maximum treatment concentration of 74.7 and 149.5 nmol/mL. In addition, TPD displayed no obvious cytotoxicity with over 90% A549 cells remaining viable under tested concentrations for 4 h.

### 3.3. Cell uptake study

As illustrated in Figure 2(B), the uptake of TPD-Rhein showed significantly higher levels than that of Rhein in A549 cells at all test concentrations (*p < .001). The intracellular amount of Rhein in A549 cells after incubation for 1 h, showed no substantial difference at 37 °C, 4 °C or in the presence of NaN_3_ (Figure 2(C)), which indicated that the uptake of Rhein was a passive diffusion process in A549 cells. By comparison, the intracellular accumulation of TPD-Rhein at 4 °C and in the presence of NaN_3_ was significantly lower than that at 37 °C, indicating a temperature- and energy-dependent uptake mechanism for TPD-Rhein. Regarding Hela cells and L929 cells, the uptake of Rhein showed a slight decrease compared with A549 cells (Figure 2(D)). However, the uptake of TPD-Rhein by these two cell lines decreased profoundly compared with that in A549 cells (Figure 2(E)), demonstrating the cell selectivity of TPD-Rhein *in vitro*.

For further mechanistic study, TPD and a series of transporter inhibitors were co-incubated with A549 cells (Figure 2(F)). Pretreatment and subsequent co-incubation of A549 cells with excess TPD, a competitive inhibitor, appeared to abolish the cellular uptake of TPD-Rhein (*p < .001, compared with control). Tetraethylammonium (TEA) chloride and imipramine as classic inhibitors of OCTs significantly reduced the uptake of TPD-Rhein, indicating the involvement of OCTs in the cell delivery of TPD-Rhein. Other selected inhibitors including pyrilamine (substrate of pyrilamine-sensitive transporter), L-carnitine (an OCTN_2_-specific substrate), choline (inhibitor of the choline transporter), and alkaline amino acids such as L-lysine, L-arginine, spermine, and spermidine, displayed no obvious impact on the cell uptake of TPD-Rhein.

### 3.4. Pharmacokinetics and biodistributions of TPD-Rhein

**Figure 3.**
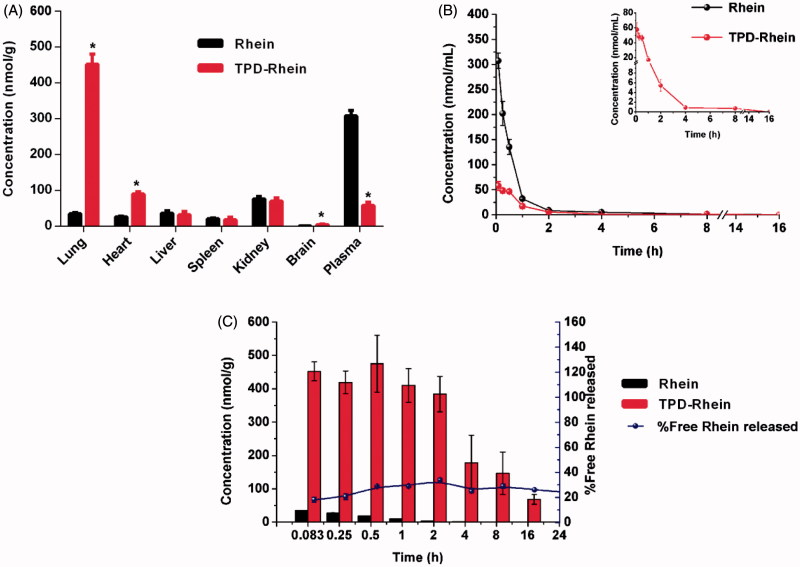
Lung-specific targeting efficiency of TPD-Rhein *in vivo*. (A) Biodistribution of Rhein and TPD-Rhein 5 min after *i.v.* injection to rats. *p < .001. (B) The mean plasma concentration-time curves of Rhein in rats after *i.v.* injection of Rhein and TPD-Rhein. (C) Lung uptake of Rhein in rats at different time points after treated with Rhein and TPD-Rhein. The concentration of TPD-Rhein includes undegraded TPD-Rhein conjugate and hydrolyzed free Rhein. Data represent mean ± SD (*n* = 5).

To evaluate the lung-targeting efficiency of TPD-Rhein, pharmacokinetics, and biodistributions of Rhein and TPD-Rhein were studied and concentration of each drug was determined by LC-MS/MS after *i.v.* injection to rats. As shown in Figure 3(A) and Figure S3, Rhein was found to accumulate mainly in kidney and plasma at 5 min after injection. Compared with Rhein, TPD-Rhein exhibited a remarkably decreased plasma concentration and a substantially increased lung accumulation at 5 min. Notably, the concentration of TPD-Rhein in the lung was the highest among all tissues tested, indicating that TPD-Rhein could selectively accumulate in the lung. The lung concentration of TPD-Rhein (451.86 ± 28.52 nmol/mL) was 13-fold that of Rhein (34.73 ± 2.82 nmol/mL, ***p < .001), while in other organs values were slightly higher or less than that of Rhein. The above results indicated a rapid and highly selective localization of TPD-Rhein in lung. Pharmacokinetic studies showed that the plasma concentration of TPD-Rhein was relatively low and was below the limit of detection after 4 h while high plasma concentration of Rhein was maintained until 12 h (Figure 3(B)). The pharmacokinetic parameters were listed in Table S1. TPD-Rhein displayed a significantly shorter half-life (t_1/2_=1.92 h) compared with Rhein (t_1/2_ = 2.39 h).

Meanwhile, the mean concentration of TPD-Rhein as well as free Rhein released from TPD-Rhein in the lung tissue was determined at the designated time points (Figure 3(C)). The concentration of TPD-Rhein was dramatically higher than that of Rhein during the time course. Free Rhein dissociated gradually from the conjugate and exhibited a similar time-concentration profile to that of TPD-Rhein. The pharmacokinetic parameters and targeting index of Rhein and TPD-Rhein were determined in major tissues tested and shown in Table 1. In contrast to the extremely low AUC_0−_*_t_* of Rhein in lung (32.24 ± 2.35 nmol/mL·h) after free Rhein treatment, TPD-Rhein significantly improved lung bioavailability of Rhein by 103-fold. In addition, calculation of targeting index including concentration efficiency (Ce) and relative uptake efficiency (Re), confirmed the profound lung targeting efficiency of TPD-Rhein with a maximum Ce_lung_ and a highest Re_lung_ among all the tissues tested.

**Table 1.** Pharmacokinetic parameters and targeting parameters of TPD-Rhein in major tissues after *i.v.* injection in rats (*n* = 5).

Parameters		AUC_(0−_*_t_*_)_ (nmol/mL·h)	MRT_0−_*_t_* (h)	*C*_max_ (nmol/mL)	CL_Z_ (L/h/Kg)	Re	Ce
Lung	Rhein	32.34 ± 2.35	0.89 ± 0.05	34.73 ± 2.82	1.09 ± 0.07	102.96	13.01
	TPD-Rhein	3329.68 ± 559.40***	6.90 ± 0.33*	451.86 ± 28.52**	0.02 ± 0.00*		
Kidney	Rhein	145.8 ± 7.64	1.77 ± 0.13	76.20 ± 6.90	0.24 ± 0.01	2.94	0.92
	TPD-Rhein	429.08 ± 24.40**	6.37 ± 0.37*	69.89 ± 9.01	0.13 ± 0.00*		
Heart	Rhein	23.17 ± 1.44	1.09 ± 0.01	7.46 ± 0.49	1.53 ± 0.09	15.80	3.41
	TPD-Rhein	366.02 ± 41.51**	7.89 ± 0.58*	25.43 ± 1.76*	0.14 ± 0.02*		
Liver	Rhein	31.76 ± 3.20	1.08 ± 0.14	36.16 ± 7.15	1.12 ± 0.13	8.90	0.88
	TPD-Rhein	282.60 ± 41.48**	5.12 ± 0.60*	31.76 ± 9.29	0.21 ± 0.03*		
Spleen	Rhein	13.06 ± 1.65	0.61 ± 0.04	20.77 ± 2.68	2.74 ± 0.32	29.02	0.86
	TPD-Rhein	379.01 ± 3.93***	8.87 ± 0.46**	17.94 ± 7.02	0.14 ± 0.02**		
Brain	Rhein	4.08 ± 0.18	2.06 ± 0.26	1.17 ± 0.20	0.86 ± 0.38	17.07	3.78
	TPD-Rhein	69.65 ± 15.10**	10.39 ± 0.71*	4.42 ± 1.11*	0.62 ± 0.19		

*C*_max_: maximum drug concentration; AUC_0−_*_t_*: area under drug concentration–time curve; MRT_0−_*_t_*: mean residence time; CL_z_: clearance rate; Ce: concentration efficiency; Re: relative uptake efficiency *p < .05, **p < .01, ***p < .001 compared with Rhein group.

### 3.5. Lung-specific targeting therapy of TPD-Rhein for asthma in rats

**Figure 4.**
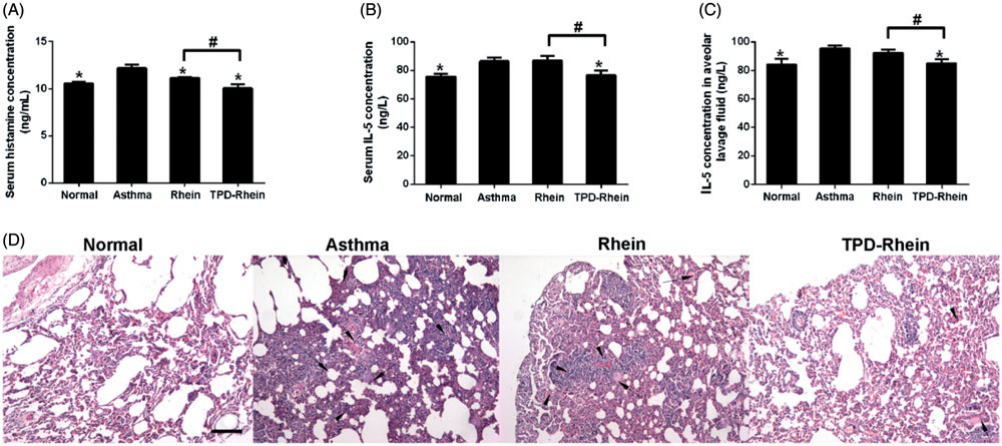
Therapeutic efficacy of TPD-Rhein on asthmatic lung in rats induced by ovalbumin. (A) Serum histamine, (B) serum IL-5 levels and (C) bronchoalveolar lavage fluid IL-5 levels of asthmatic rats after TPD-Rhein treatment. (D) Histological analysis showing TPD-Rhein ameliorated inflammation manifested in lung. Data represent mean ± SD (*n* = 5). *p < .05, compared with asthma group. #*p* < .05. Scale bar =100 μm.

The *in vivo* therapeutic efficiency of TPD-Rhein was evaluated in a rat asthma model challenged by OVA. As shown in Figure 4(A), TPD-Rhein treatment significantly inhibited plasma histamine level (a mast cell-specific mediator involved in asthma, Figure 4(A)) in a timely manner, compared to the control groups (asthmatic model group and Rhein treated group). Moreover, IL-5 levels (a marker of the eosinophilic inflammatory process in asthma, Figure 4(B)) in plasma as well as bronchoalveolar lavage fluid markedly decreased during the course of asthma after TPD-Rhein treatment (Figure 4(B,C)), as compared with asthmatic model rats without any treatment. In contrast, no significant differences were observed between the IL-5 levels of Rhein treated group and asthmatic model group. Histological examinations on lung sections of asthmatic model rats demonstrated lymphoid infiltration and thickening of alveolar septa throughout the parenchyma (Figure 4(D)), in comparison with those of normal group. TPD-Rhein treatment notably attenuated tissue damage characterized by induced pulmonary alveolar thickening, decreased inflammatory cell infiltration and mild bronchial epithelial hyperplasia while free Rhein group displayed no significant improved anti-asthma efficacy compared with asthmatic model rats. These results suggested that TPD-Rhein showed stronger inhibition on the asthmatic inflammation manifested in lung compared with free Rhein.

### 3.6. *In vivo* toxicity of TPD-Rhein

**Figure 5.**
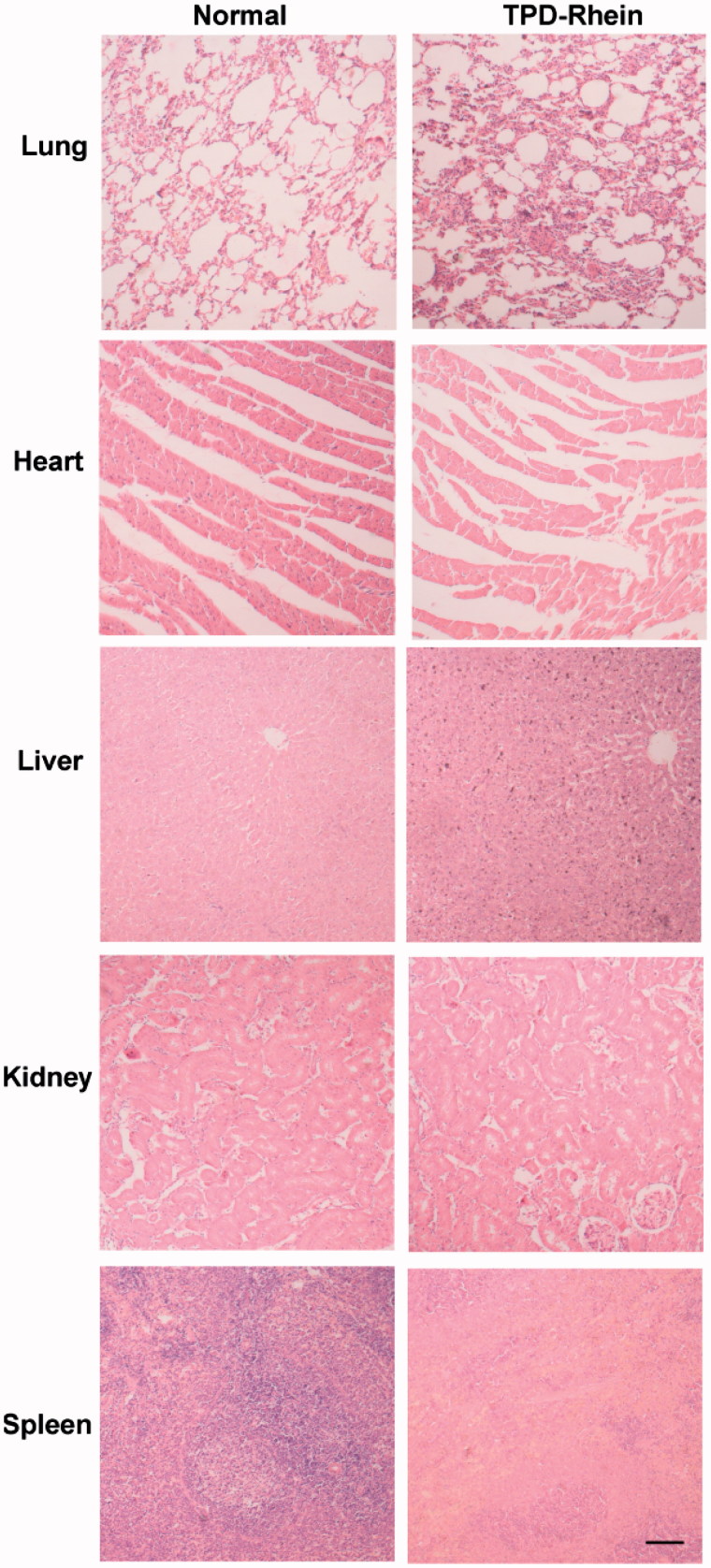
*In vivo* toxicity evaluation of TPD-Rhein in rats. The major tissues were processed for H&E staining 24 h after the last treatment of TPD-Rhein. No apparent changes were observed in these tissues. Scale bar =100 μm.

Toxicity remains another critical factor to evaluate the application of potential therapeutic agents. H&E staining of major organs revealed no inflammation or significant changes induced by TPD-Rhein under the designed dosing regimen (Figure 5). These results indicated the safety of TPD as a lung-targeting ligand.

## 4. Discussions

Despite the great potential for developing novel therapeutics for asthma, lung-targeted drug delivery *via* systemic administration faces formidable hurdles before it can make a substantial progress. Most studies focused on utilizing nanoplatforms to enhance pulmonary drug accumulation but their applications were limited by low targeting efficiency, materials-related toxicity, complex manufacturing processes as well as poor batch-to-batch reproducibility (Shi et al., 2013; Shi et al., 2016). Little attention has been paid to small ligands-based prodrug approach, which has definitive structure, avoid material-related toxicity and thus represents a remarkable tool for clinical applications. More efforts are therefore needed to establish its role in asthma therapy. Based on the previously reported lung-imaging agent HIPD (Miniati et al., 1996), we developed a new HIPD analog to afford TPD-Rhein through ester bond formation and exhibited outstanding lung-targeting efficiency as well as remarkable anti-asthmatic activity.

From a simple design concept, the proposed ligand TPD was synthesized from commercially available compounds in two-step reaction, which was more convenient than the conventional synthesis of HIPD through four steps (Moerlein et al., 1990). TPD with phenolic propanediamine at the *para*-position reduced the steric hindrance around the phenol hydroxyl group and thereby was readily conjugated to Rhein. Regarding the stability in physiological conditions, TPD-Rhein gradually degraded as the incubation time increased in varying pH values of PBS without esterase (Figure 2(B)). In contrast, TPD-Rhein degraded faster in plasma after 10 min incubation, probably due to the hydrolysis of phenyl ester bond by plasma esterase. It should be noted that the degradation did not impair the targeting effect of TPD-Rhein *in vivo*, as revealed by biodistribution study. TPD-Rhein exhibited 12-fold higher drug concentration in lung tissues than Rhein at 5 min after intravenous administration, indicating its rapid and efficient accumulation in lung. In addition, TPD-Rhein showed the highest Re in the lung among all major tissues, indicating that the proposed phenolic propanediamine-based moiety had good lung-targeting ability, which confirmed our hypothesis. Meanwhile, free Rhein dissociated gradually from the conjugate and exhibited a similar time-concentration profile to that of TPD-Rhein in lung. This was in accord with the results obtained from *in vitro* hydrolysis assays showing TPD-Rhein exhibited sustained release properties in lung homogenates for 16 h (Figure 1(D)). All these results strongly suggested that TPD could gradually deliver a sufficient quantity of active agents to lung *via* simple hydrolysis of ester bonds to obtain sustained therapeutic effect.

We next evaluated the *in vitro* cytotoxicity of TPD by standard MTT assay in the representative A549 cell line (human alveolar epithelial cells) (Keenan et al., 2012). The results indicated that the lung-targeted ligand TPD was minimally cytotoxic, which was essential for the design of safe conjugates. More importantly, TPD-Rhein exhibited substantially higher uptake into A549 cells and superior cell selectivity than Rhein, as revealed by cellular uptake studies (Figure 2(A)). Regarding this, we attempted to elucidate the underlying cellular uptake mechanisms. The uptake of TPD-Rhein by A549 cells was temperature- and energy-dependent (Figure 2(E)). Organic cation transporters (OCTs) were found to play a major role in mediating cell uptake of Rhein by TPD (Figure 2(F)).

Along with its good lung-targeting efficiency, TPD-Rhein exhibited significantly lower concentration and shorter half-life than Rhein in plasma, as demonstrated by pharmacokinetic studies (Figure 3(B)). It is well known that nanocarriers-based targeting approaches require long plasma half-life due to passive accumulation by the enhanced permeability and retention (EPR) effect (Lyer et al., 2006). Our findings with the systemic delivery of TPD-Rhein conjugate differ from that of nanocarriers-based targeting approaches. We hypothesized that the rapid accumulation of TPD-Rhein in lung may be orchestrated in a hierarchical manner and in turn shorten its plasma half-life. First, some specific transporters expressed within lung, such as OCTs, may actively transport TPD-Rhein into lung. Second, the esterification of Rhein may increase its lipophilicity, leading to efficient cell permeability. Additionally, with the presence of tertiary amino groups, TPD-Rhein may be readily retained in the lung by pre-systemic extraction through interaction with negative cell contents (Bend et al., 1985). As a new lung-targeting moiety, studies are underway to further elucidate specific mechanism behind the lung targetability of TPD-Rhein.

The *in vivo* therapeutic efficacy was well defined by OVA-induced asthma model in rats. Histamine is a major mediator that induces a series of acute pathological responses in asthma (Dunford & Holgate, 2010). IL-5, a type-2 cytokine, plays a crucial role in the initiation and growth of eosinophilic airway inflammation in asthma (Pelaia et al., 2012). These two key indicators for asthma were therefore determined to evaluate the efficacy of TPD-Rhein *in vivo*. Systemically administered TPD-Rhein conjugate was proven to protect lungs from damages in a timely manner and suppress inflammatory responses in the lungs through decreasing plasma histamine level, plasma IL-5 level as well as alveolar IL-5 levels. Conversely, free Rhein lacked lung-specific localization and resulted in poor efficacy in asthmatic rats (Figure 4). These results in combination with attenuated inflammation in lung tissues observed by histological evaluation, suggested that TPD-Rhein conjugate successfully decreased the severity of asthma inflammation, attributing to good lung-targeting performance.

On the basis of these results, we proposed the following process for TPD mediated lung delivery of Rhein to treat asthma. Once injected into rat tail vein, TPD-Rhein could cause an immediate accumulation in the lung, thus avoiding quick hydrolysis by plasma enzyme. Subsequently, TPD-Rhein demonstrated highly efficient cellular uptake within lung alveolar cells mainly mediated by OCTs. In the end, a simultaneous dissociation of TPD-Rhein triggered by lung enzymes gradually releases Rhein as in free form to treat inflammation in asthmatic lung. Unfortunately, there was still a small proportion of Rhein that was released to plasma before reaching target organ as a time-dependent degradation of TPD-Rhein was found in incubation with plasma (Figure 1(C)).

While this study validates the lung-targeting efficiency and therapeutic efficacy of TPD-Rhein, additional studies are necessary to further optimize the new lung-targeting ligand TPD for clinical translation. Further studies are also planned to expand the approach to other therapeutic agents for the treatment of lung-related diseases.

## 5. Conclusions

In sum, TPD as a new lung-targeting ligand successfully tailored an anti-inflammatory compound Rhein to preferentially accumulate in lung with rapid distribution kinetics and excellent targeting efficiency. Accordingly, drug-ligand conjugate effectively inhibited inflammation of asthma in rats. To our best knowledge, this is the first report showing a lung-specific targeting strategy based on a phenolic propanediamine moiety for efficient asthma therapy. Our study therefore may contribute to the development of lung-targeted drug delivery to improve the efficacy of additional therapeutics for lung-related diseases.

## Supplementary Material

Supplemental Material
