# Supplementary material for: A novel phenolic propanediamine moiety-based lung-targeting therapy for asthma
Source: Drug Deliv. 2018 May 21;25(1):1117–26. doi: 10.1080/10717544.2018.1472675 (PMC6058525; doi:10.1080/10717544.2018.1472675)
Supplement: Supplemental Material [file IDRD_A_1472675_SM5405.doc]

**Supplemental material**

**A novel phenolic propanediamine moiety-based lung-targeting therapy for asthma**

*Jianbo Li^1^, Yang Yang^1^, Didi Wan^1^, Youmei Peng^1^, Jinjie Zhang^2,3,4^**

^1^ Institute of Medical and Pharmaceutical Sciences, Zhengzhou University, *No.40. Daxue Road,* Zhengzhou, Henan Province 450052, China.

^2^ School of Pharmaceutical Sciences, Zhengzhou University, *No.100. Kexue Road,* Zhengzhou, Henan Province 450001, China.

^3^ Key Laboratory of Targeting Therapy and Diagnosis for Critical Diseases, Zhengzhou 450001, PR China

^4^ Collaborative Innovation Center of New Drug Research and Safety Evaluation, Henan Province, People’s Republic of China

* Address for correspondence: Jinjie Zhang. School of Pharmaceutical Sciences, Zhengzhou University, *No.100. Kexue Road,* Zhengzhou, Henan Province 450001, China. Tel: +86-0371-67781908. E-mail: liger1029@126.com.

Table S1. Pharmacokinetic parameters of Rhein and TPD-Rhein in plasma after *i.v.* injection in rats (n=5)

| Drugs | AUC_(0-t)_ (nmol/mL·h) | t_1/2_ (h) | C_max_ (nmol/mL) | CL_Z_ (L/h/Kg) | V_Z_ |
| --- | --- | --- | --- | --- | --- |
| Rhein | 205.97± 10.59 | 2.39 ± 0.09 | 307.50 ± 3.89 | 0.17 ± 0.87 | 0.47± 0.02 |
| TPD-Rhein | 65.59 ± 5.49* | 1.92± 0.06* | 57.71± 7.99* | 0.93 ± 0.08* | 3.21± 0.40* |

**p* < 0.05, compared with Rhein.

Table S2 Aqueous Solubility of Drugs (n=3)

| Drug | Solubility (mg/mL) | |
| --- | --- | --- |
|  | pH 2.0 | pH 7.4 |
| Rhein | 0.00356±0.0002 | 0.513±0.032 |
| TPD-Rhein | 13.034±0.71 | 0.00472±0.0005 |


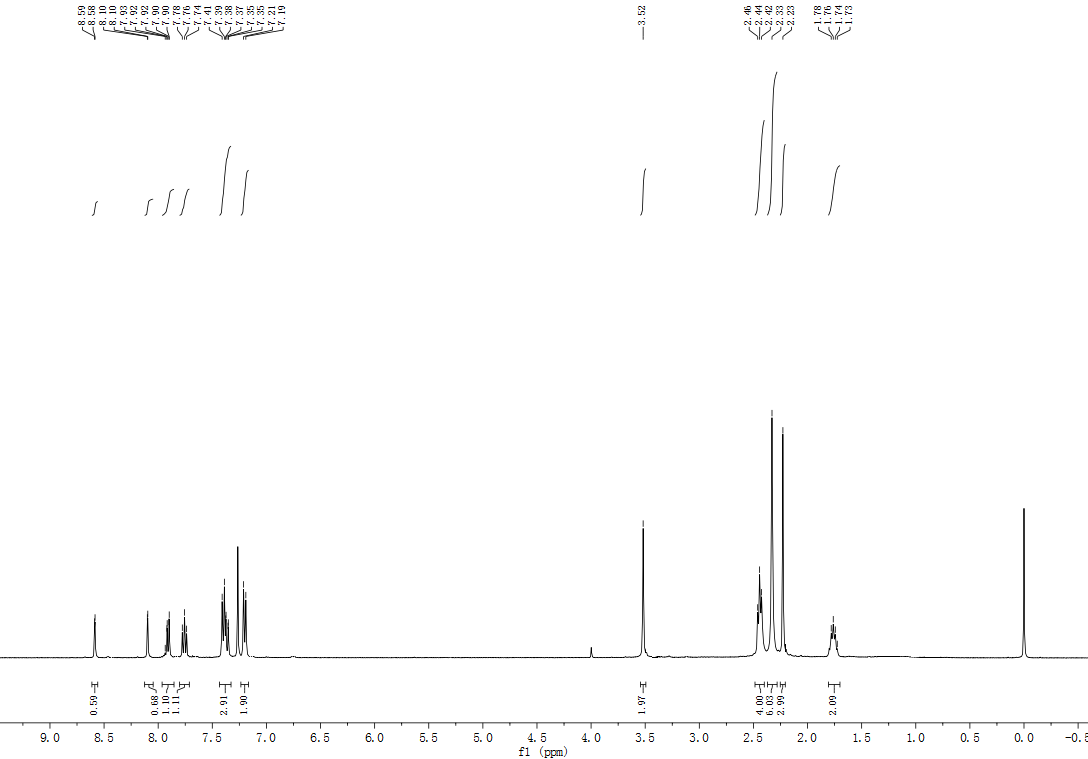


Figure S1. ^1^H-NMR spectra of TPD-Rhein

**
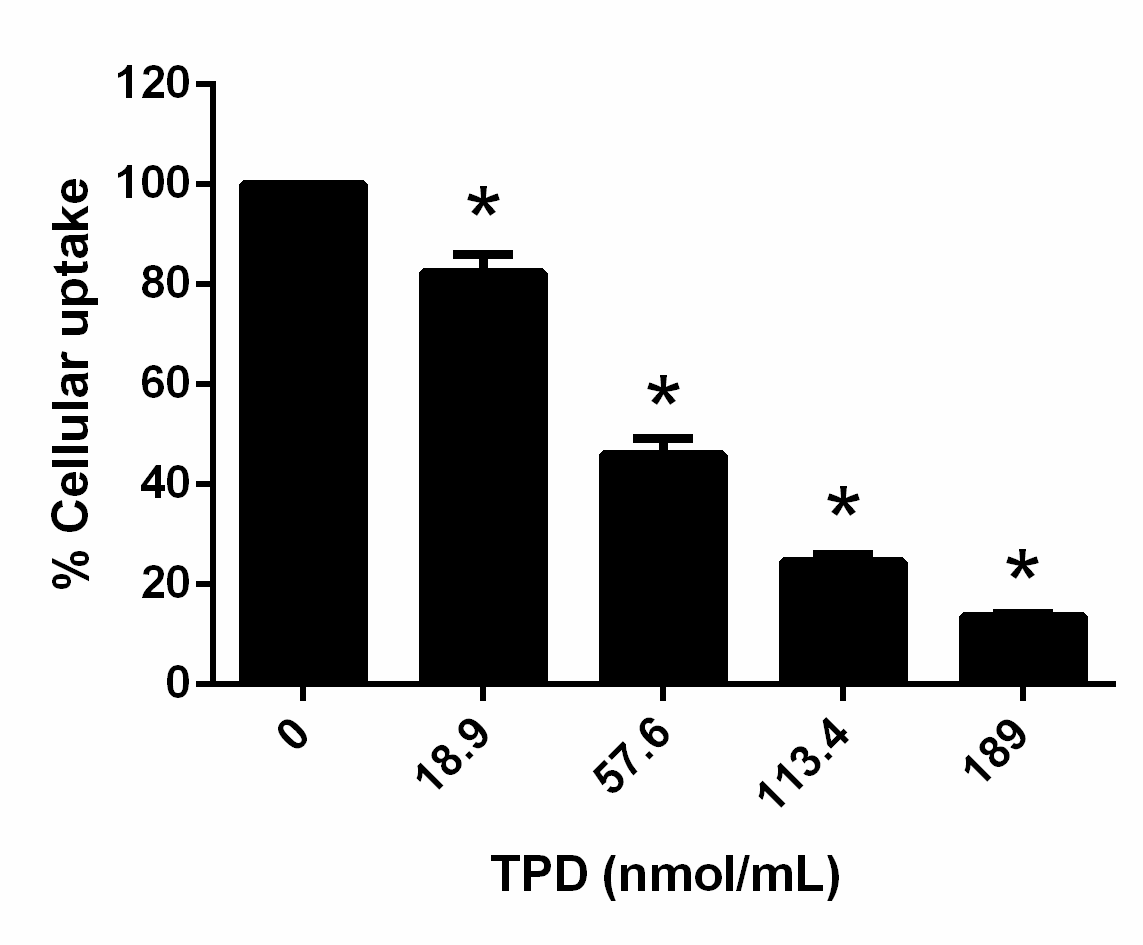
**

Figure S2. Competitive inhibition of TPD ligand on the cellular delivery efficiency of TPD-Rhein conjugate by A549 cells. Data represent as mean ± S.D. (n = 3). ^*^*p <* 0.001, compared with the cellular uptake efficiency of TPD-Rhein without TPD inhibition treatment.


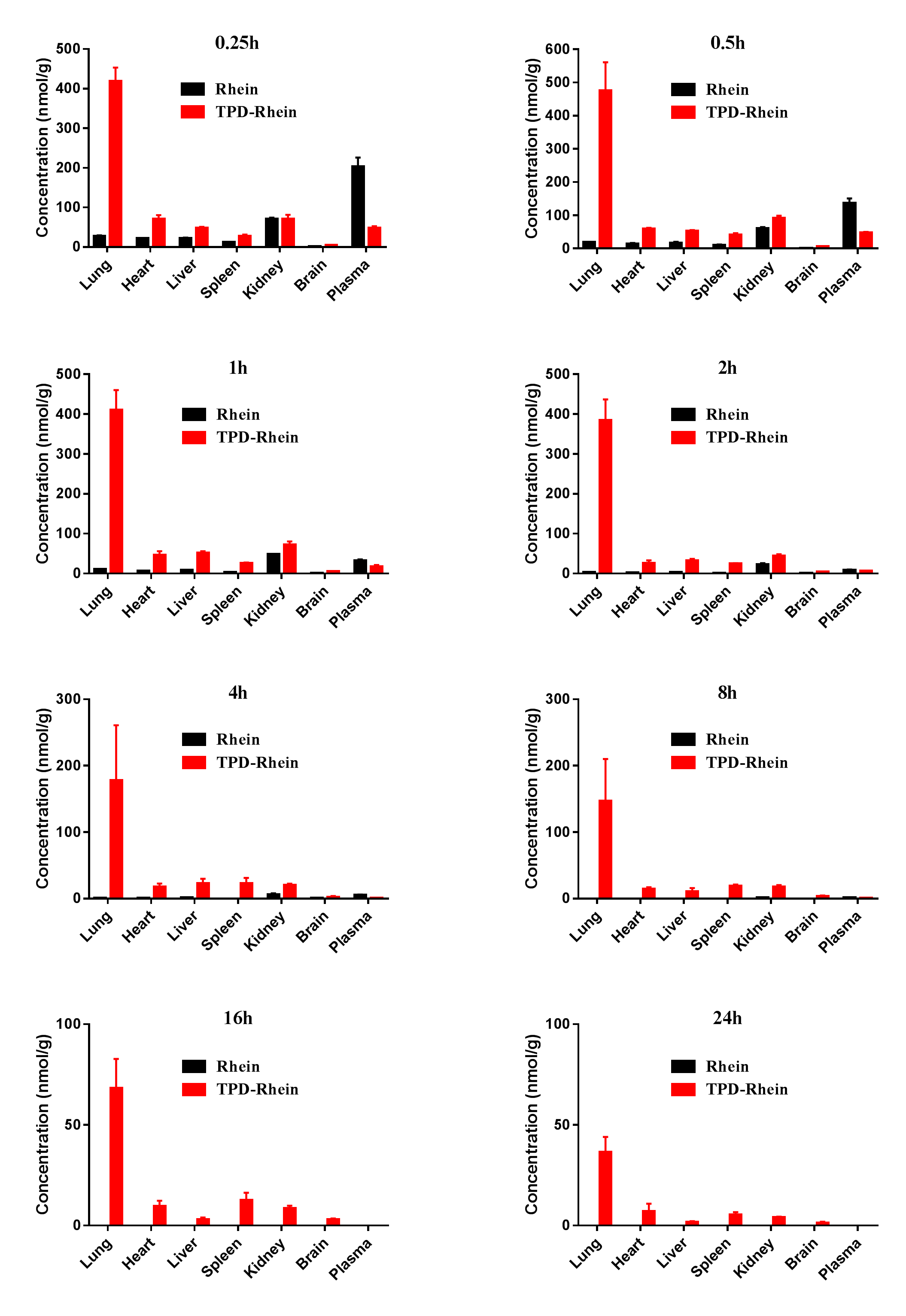


Figure S3 Biodistribution profiles of Rhein and TPD-Rhein following i.v. injection at a Rhein-equivalent dose of 10 mg/kg in rats. The concentrations of TPD-Rhein were converted to Rhein equivalent. Data represented as mean ± SD (n=5).
